# Protein Residues and a Novel Motif Involved in the Cellular Localization of CheZ in *Azorhizobium caulinodans* ORS571

**DOI:** 10.3389/fmicb.2020.585140

**Published:** 2020-12-07

**Authors:** Xiaolin Liu, Yanan Liu, Kevin Scot Johnson, Xiaoyan Dong, Zhihong Xie

**Affiliations:** ^1^Key Laboratory of Coastal Environmental Processes and Ecological Remediation, Yantai Institute of Coastal Zone Research, Chinese Academy of Sciences, Yantai, China; ^2^College of Resources and Environment, University of Chinese Academy of Sciences, Beijing, China; ^3^Department of Microbiology and Environmental Toxicology, University of California, Santa Cruz, Santa Cruz, CA, United States; ^4^Center for Ocean Mega-Science, Chinese Academy of Sciences, Qingdao, China; ^5^National Engineering Laboratory for Efficient Utilization of Soil and Fertilizer Resources, College of Resources and Environment of Shandong Agricultural University, Taian, China

**Keywords:** chemotaxis, CheZ, cellular localization, *Azorhizobium caulinodans*, rhizobia

## Abstract

Chemotaxis is essential for the competitiveness of motile bacteria in complex and harsh environments. The localization of chemotactic proteins in the cell is critical for coordinating a maximal response to chemotactic signals. One chemotaxis protein with a well-defined subcellular localization is the phosphatase CheZ. CheZ localizes to cell poles by binding with CheA in *Escherichia coli* and other enteric bacteria, or binding with a poorly understood protein called ChePep in epsilon-*Proteobacteria*. In alpha-*Proteobacteria*, CheZ lacks CheA-binding sites, and its cellular localization remains unknown. We therefore determined the localization of CheZ in the alpha-*Proteobacteria Azorhizobium caulinodans* ORS571. *A. caulinodans* CheZ, also termed as CheZ_AC,_ was found to be located to cell poles independently of CheA, and we suspect that either the N-terminal helix or the four-helix bundle of CheZ_AC_ is sufficient to locate to cell poles. We also found a novel motif, AXXFQ, which is adjacent to the phosphatase active motif DXXXQ, which effects the monopolar localization of CheZ_AC_. This novel motif consisting of AXXFQ is conserved in CheZ and widely distributed among *Proteobacteria*. Finally, we found that the substitution of phosphatase active site affects the polar localization of CheZ_AC_. In total, this work characterized the localization pattern of CheZ containing a novel motif, and we mapped the regions of CheZ_AC_ that are critical for its polar localization.

## Introduction

In harsh and complex environments, bacteria must adapt and respond to external changes quickly. Chemotaxis systems are one-way bacteria have envolved to do this. Chemotaxis enables bacteria to regulate their motility in response to environmental signals. The chemotaxis pathway has been well studied in *Escherichia coli*. External signals or nutrient molecules are sensed by chemoreceptors. Upon binding with attractant signals, conformational changes of chemoreceptors inhibit the autokinase activity of the associated histidine kinase CheA. In the presents of a repellent signal, CheA can phosphorylate the response regulator CheY, and CheY-P diffuses and binds with the flagellar motor proteins FliM and FliN, causing the flagella to change rotational direction from counterclockwise to clockwise (Szurmant and Ordal, 2004). The phosphatase CheZ promotes the intrinsic dephosphorylation of CheY-P to terminate the signal transduction (Blat and Eisenbach, 1994; Silversmith et al., 2008; Silversmith, 2010).

The spatial organization of chemotaxis proteins is critical for bacterial chemotaxis to adapt to environments. Chemotaxis proteins are localized to cellular poles using multiple strategies, including the nucleoid occlusion, Tol/Pal complex, membrane curvature, and protein-protein interactions (Laloux and Jacobs-Wagner, 2014). Transmembrane *E. coli* chemoreceptors maintain polar localization through the Tol/Pal complex, strong membrane curvature, or nucleoid exclusion (Santos et al., 2014; Neeli-Venkata et al., 2016; Saaki et al., 2018) The Tol/Pal complex is a conserved component of bacterial cell envelope, which is involved in the maintenance of cell wall integrity (Bernadac et al., 1998). Other chemotaxis proteins including CheA, CheW, CheY, and CheZ locate to cellular poles based on the interaction with other chemotaxis proteins (Sourjik and Berg, 2000). CheA and CheW can bind to chemoreceptor forming polar chemotaxis complexes (Pinas et al., 2016), and the localization of CheZ and CheY depends on the presence of CheA in *E. coli* (Sourjik and Berg, 2000).

CheZ is encoded in around 40% of bacterial genomes (Wuichet and Zhulin, 2010), and the localization mechanism of CheZ has been well studied in *E. coli*. *E. coli* CheZ, termed as CheZ_EC_, locates to cellular poles with the help of CheA-short (CheAs), a short form of CheA lacking the first 97 amino residues of full length CheA, called CheA-long. CheZ_EC_ interacts with CheA using a small region of amino acids with most interactions coming from the apical hairpin loop consisting of two aromatic residues, Phe-97 and Trp-98 (Cantwell and Manson, 2009). For CheAs, two hydrophobic residues Leu-123 and Leu-126 in the N-terminus of CheA are responsible for CheZ_EC_ interactions (Cantwell et al., 2003; Hao et al., 2009).

*Azorhizobium caulinodans* ORS571 is a rhizobium belonging to alpha-*Proteobacteria* uses chemotaxis for plant colonization. It fixes nitrogen with the host *Sesbania rostrata* by forming stem or root nodules (Dreyfus et al., 1983). *A. caulinodans* ORS571 has only one chemotaxis pathway including one gene cluster (*cheA*, *cheW*, *cheY2*, *cheB*, and *cheR*) and two orphan genes (*cheY1* and *cheZ*) (Jiang et al., 2016). Deletion of one or several genes within the *A. caulinodans* ORS571 chemotaxis cluster reduces or abolishes the chemotaxis of *A. caulinodans* ORS571, confirming the role of these genes in chemotaxis (Liu W. et al., 2018; Liu X. et al., 2018). Deletion of *cheZ* causes *A. caulinodans* non-chemotactic, while in contrast to other chemotaxis proteins which are important for host plant colonization, CheZ plays negative roles on early colonization (Liu et al., 2019). We previously found that a soluble heme-binding chemotaxis protein in *A. caulinodans* locates at the cell poles with the help of CheA (Jiang et al., 2016). However, it has been reported that CheZ proteins in alpha- and delta-*Proteobacteria* lack the sequences responsible for CheAs binding (Wuichet et al., 2007), and the localization of CheZ in alpha-*Proteobacteria* remains unknown.

In the present study, we reported the localization pattern of CheZ in *A. caulinodans* ORS571 and mapped regions of CheZ_AC_ that are sufficient for polar localization by constructing various truncated mutants of CheZ_AC_. Furthermore, a novel motif in CheZ_AC_, which is conserved among *Proteobacteria*, was found to be involved in the regulation of monopolar CheZ localization.

## Results

### Bioinformatics Analysis Shows that CheZ_AC_ Lacks Canonical Sites Involved in CheZ_EC_ Polar Localization

The structure of CheZ_EC_ consists of four regions, including an N-terminal helix (residues 1–34) of unknown functions, a four-helix bundle formed from a dimer of two hairpin structures (residues 35–168), a linker (residues 169–199), and a C-terminal helix (residues 199–214) (Zhao et al., 2002; Silversmith, 2005). Because the amino acid sites involved in the CheZ_EC_ polar localization were well studied, we first aligned the CheZ amino acid sequences from *E. coli* (CheZ_EC_) and *A. caulinodans* (CheZ_AC_) using an EMBOSS Needle program (Madeira et al., 2019). Then, we modeled the structure of CheZ_AC_ using online server SWISS-MODEL (Waterhouse et al., 2018) and Jpred4 (Drozdetskiy et al., 2015).

**FIGURE 1:**
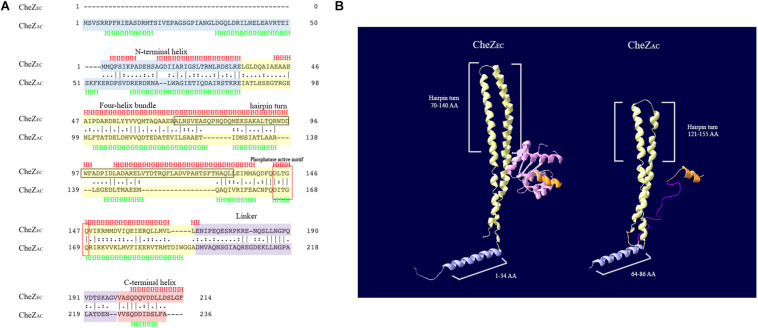
Bioinformatics analysis of CheZ_EC_ and CheZ_AC_. **(A)** The alignment of CheZ_EC_ and CheZ_AC_ was generated using the default settings of EMBOSS Needle software from EMBL. The N-terminal helix, four-helix bundle, linker, and C-terminus of CheZs were marked with blue, yellow, purple, and red color, respectively. The region containing hairpin turn, which is sufficient for localization of CheZ_EC_, was marked with black frame. The secondary structure was predicted with Jpred4 program. **(B)** Structure overview of CheZ_EC_ (1KMI) and CheZ_AC_. The N-terminal helix, four-helix bundle, linker, and C-terminus of CheZs were recolored as above. CheY (marked with pink) was shown in the structure of CheZ_EC_ and the linker region was not shown in CheZ_EC_. The structure model of CheZ_AC_ was produced by SWISS-MODEL program, and the first 50 amino acids (involved in its N-terminal helix) were not shown.

An alignment of CheZ_AC_ and CheZ_EC_ proteins showed significant similarity (33.9%) between them and both of them have conserved phosphatase active sites (Asp 165 and Gln 169 in CheZ_AC_) (Figure 1A), which is consistent with our previous report (Liu X. et al., 2018). Structure modeling results (Figures 1A,B) suggested CheZ_AC_ also has the N-terminal helix (residues 1–86 in CheZ_AC_), four-helix bundle hairpin (residues 87–195 in CheZ_AC_), the linker (residues 196–225 in CheZ_AC_), and the C-terminal helix (residues 225–236 in CheZ_AC_). Interestingly, the N-terminal helix in CheZ_AC_ is substantially longer (∼50 residues) than CheZ_EC_, while the four-helix bundle hairpin (∼25 residues) in CheZ_AC_ is substantially shorter than CheZ_EC_ (Figure 1). And, the gaps in the alignment are similar in size on either side of the hairpin turn (residues ∼140), which is consistent with a shorter bundle (Figures 1A,B). Remaining of residues from 70 to 133 including the tip of hairpin (residues ∼100) is sufficient for polar localization of CheZ_EC_ (Cantwell and Manson, 2009). The similarity of the hairpin tip between CheZ_AC_ and CheZ_EC_ indicates that the hairpin tip might be also employed by CheZ_AC_ to bind potential localization partner proteins. However, a conserved motif D(D/E)WF (residues 95–98) (Cantwell et al., 2003), which is important for CheZ_EC_ polar localization, was not found in CheZ_AC_ (Figure 1A).

In *E. coli*, the polar localization of CheZ is achieved by binding with a short form of CheA (CheA_EC_), which begins at Met-98 of full length CheA_EC_ (Cantwell et al., 2003; Hao et al., 2009). There is only one CheA protein encoded in *A. caulinodans* genome, termed as CheA_AC_. When we made a pairwise sequence alignment of CheA_EC_ and CheA_AC_, the absence of cognate CheA_EC_ Met-98 in CheA_AC_ suggests that CheA_AC_ does not have a short form of CheA (Supplementary Figure S1). These results suggest that CheZ_AC_ may not locate to cell poles or locate to cell poles with a different mechanism from CheZ_EC_.

### Characteristics of CheZ Localization in *A. caulinodans* ORS571

**FIGURE 2:**
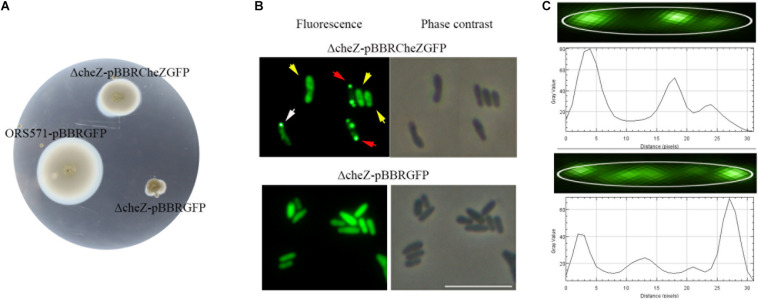
Cellular localization of CheZ in *A. caulinodans* ORS571. The plasmid pBBR2CheZ-GFP containing native promoter can restore the chemotaxis deficiency of *cheZ* mutant **(A)**. The sublocalization of CheZ_AC_ is heterogenous including diffuse (indicated with yellow arrows), monopolar (indicated with white arrows), and bipolar (red arrows) localization **(B)**. The spatial distribution of CheZ clusters **(C)**, and the edge of cell body was outlined with white line. The gray value peaks correspond to the GFP spots of CheZ clusters. The length of the cell matched with the length of the *x*-axis. The scale bar in **panel B** represents 10 μm.

To study the subcellular localization of CheZ in *A. caulinodans* cells, we designed a C-terminal GFP fusion to CheZ_AC_. To avoid artifacts related overexpression, the expression of the fusion gene was controlled by the native promoter of *cheZ* (Liu X. et al., 2018). When the CheZ_AC_ fusion was expressed in the *cheZ* mutant strain, the chemotactic behavior of the *cheZ* null mutant was partially complemented to wild-type levels that contains a control vector pBBR2GFP, indicating the CheZ-GFP retains function (Figure 2A). Another evidence showing that CheZ-GFP functions is that the presence of CheZ-GFP restores Δ*cheZ* flagella rotating to wild-type level. Wild type and Δ*cheZ* complemental strains both rotate their flagella between clockwise and counter-clockwise, while Δ*cheZ* with or without pBBR2GFP always shows counter-clockwise rotation (Unpublished data).

**FIGURE 3:**
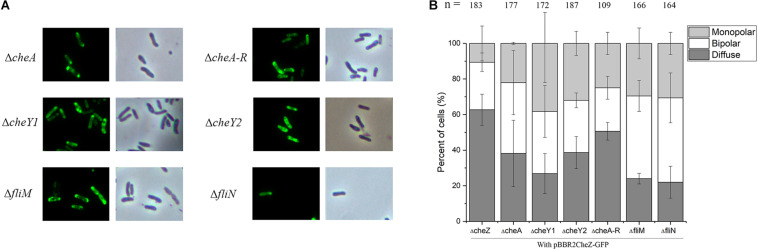
The localization pattern of CheZ in *cheA*, *cheA-R*, *cheY1*, *cheY2*, *fliM*, and *fliN* mutant. The full-length CheZ including its upstream promoter was fused to GFP, and the plasmid containing CheZ-GFP was introduced into each mutant. **(A)** Representative images of CheZ_AC_ localization in each strain. **(B)** Quantification of cells ratios showed different localized pattern of CheZ_AC_. Values are shown as the means and standard deviations from at least three independent experiments.

We next determined the spatial distribution of CheZ-GFP by fluorescence microscopy. The CheZ-GFP fusion showed three unique localization patterns (Figure 2B). We manually determined and quantified these localization patterns using ImageJ, comparing the brightness of polar foci with that of cell body (see Materials and Methods). About 60% of cells demonstrated diffuse CheZ-GFP localization, 30% of cells CheZ-GFP localized to both cell poles, and 10% of cells showed monopolar localization (Figure 3B). The percent of cells with polar localized CheZ in *A. caulinodans* are significantly lower than that in *E. coli*, in which CheZ_EC_ shows polar localization in 85% of the cells (Blat and Eisenbach, 1996). Except polar and diffuse localized pattern, there were also many lateral clusters of CheZ-GFP (Figure 2C). When analyzing the distance from each lateral cluster to the pole of cell, the position of each lateral cluster distributed along the cell body with a period corresponding to the 1/2 or 1/4 of the cell length (Figure 2C).

### The Polar Localization of CheZ_AC_ Is Independent of Chemotaxis and Flagellar Proteins

Numerous studies have shown that chemotaxis protein can form a polar cluster to better adapt to environmental conditions. To study whether the polar localization of CheZ_AC_ is dependent on CheA or other chemotaxis proteins, we examined the localization patterns of CheZ_AC_ in different backgrounds lacking different chemotaxis proteins. Consistent with our bioinformatics analysis, deletion of *cheA* does not alter the cellular localization of CheZ_AC_ (Figure 3). We then tested the localization pattern of CheZ_AC_ in the following chemotaxis mutants, Δ*cheY1*, Δ*cheY2*, or Δ*cheA-R* clusters (including *cheA*, *cheY2*, *cheW*, *cheB*, and *cheR*) (Liu W. et al., 2018; Liu et al., 2020). Interestingly, CheZ_AC_ maintains polar localization in both the Δ*cheY1*, Δ*cheY2*, and Δ*cheA-R* mutants backgrounds (Figure 3).

Next, we tested if flagellar proteins affect CheZ_AC_ polar localization. FliM and FliN are two flagellar motor components and interact with CheY either directly or indirectly (Delalez et al., 2014). Deletion of either one makes *A. caulinodans* non-flagellated and non-motile (Shen et al., 2018), however, neither of them abolished the polar localization of CheZ_AC_ (Figure 3). These results indicate that CheZ_AC_ is localized to the cell poles independent of chemotaxis or flagellar proteins.

### N-terminal Helical Regions Are Sufficient for CheZ_AC_ Polar Localization

**FIGURE 4:**
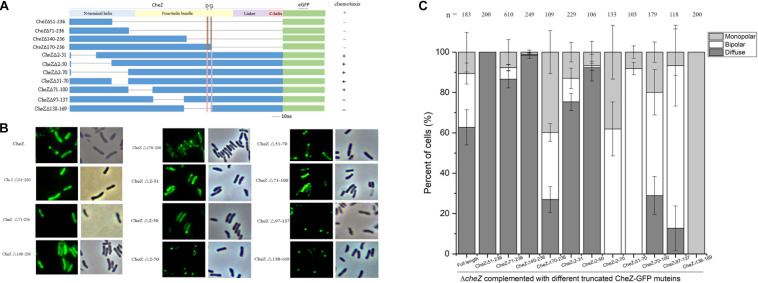
The regions of CheZ involved in the polar localization. The overview of the deleted region in CheZ_AC_ and their effects on chemotaxis **(A)**. The truncated CheZ proteins were fused to green fluorescent protein at the carboxyl-terminal ends. The letter “D” and “Q” above the figure indicate the position of the conserved phosphatase motif, DXXXQ, of CheZ. The representative images and quantification of the localized patches of various truncated CheZ in Δ*cheZ* background **(B,C)**. The “+” and “-” in **panel A** means strain containing cognate CheZ derivatives can or cannot restore chemotaxis. Values are expressed as means ± standard deviation from at least three independent experiments.

Although there is low conservation between *E. coli* and *A. caulinodans* CheZ, the C-terminal sequences including CheY-P binding region and phosphatase active sites are conserved (Blat and Eisenbach, 1996; Zhao et al., 2002; Wuichet et al., 2007; Silversmith, 2010; Liu X. et al., 2018). The N-terminal helix, whose function remains unknown, and the middle four-helix bundle hairpin of CheZ, which is required for localization in *E. coli*, are variable (Lertsethtakarn and Ottemann, 2010). To map the region sufficient for polar localization, various portions of the N-terminal helix (residues 1–86), and middle four-helix bundle regions (residues 87–195) of CheZ_AC_ were fused to GFP (Figure 4A). CheZΔ51-236, containing a portion of N-terminal helix of CheZ_AC_, failed to localize to the cell poles (Figures 4B,C). Surprisingly, CheZΔ71-236, containing nearly all regions of the N-terminal helix can locate to cellular poles, though the number of cells with bipolar localized CheZ_AC_ decreased from 30 to 5% compared to full-length CheZ_AC_ (Figures 4B,C). Because the four-helix bundle, especially its hairpin tip, is essential for the polar localized pattern of CheZ_EC_ (Hao et al., 2009), we made a longer truncated mutant containing a portion of the four-helix bundle CheZΔ140-236. Intriguingly, CheZΔ140-236 can localize to cell poles, however, the cell ratio showed polar localized pattern decreased no more than 5%. When the remaining residues extended from 1–139 to 1–169, including almost all the region of N-terminal helix and four-helix bundle, the CheZΔ170-236 mutant can locate to mono- or bi-polar poles in cells above 70% (Figure 4). These results suggest that the role of N-terminal helix and four-helix bundle on the polar localization of CheZ_AC_ is different from that of CheZ_EC_. The N-terminal helical region is sufficient for polar localization of CheZ_AC_, and the four-helix bundle is involved in the regulation of CheZ polar localization.

To further determine the regions of CheZ that are responsible for the polar localization in *A. caulinodans*, CheZ_AC_ proteins with various deletions at the N-terminal helix and four-helix bundle were fused to GFP (Figure 4A). All the fusion proteins were introduced into the *cheZ* mutant and expressed with the native promoter. When part of the CheZ_AC_ N-terminal helix was deleted, including residues from 2 to 31 or from 2 to 50 (termed as CheZΔ2-31 and CheZΔ2-50), the truncated mutant maintained polar localization, though the polar localized CheZ_AC_ decreased from 40 to 25, and 10%, respectively (Figures 4B,C). Unexpectedly, deletions of N-terminal helical regions from residues 2–70 (CheZΔ2-70) or 51–70 (CheZΔ51-70) did not abolish the polar localization of CheZ_AC_ (Figures 4B,C). These results suggest that the region from residues 2–70 might not be the sole region sufficient for polar localization of CheZ_AC_. We further tested the polar localized pattern of mutants lacking part of the four-helix bundle hairpin, CheZΔ71-100, CheZΔ97-137, and CheZΔ138-169, and we found that all of them remained the polar location of CheZ_AC_. These results suggest that CheZ_AC_ might be anchored to cell poles via multiple motifs. Interestingly, CheZΔ138-169 not only maintains polar localization, but also shows 100% monopolar localized pattern (Figure 4).

### Mining for a Novel Motif Involved in the Regulation of CheZ_AC_ Localization

We next sought to identify what protein or residues are responsible for the unique monopolar localization pattern of CheZΔ138-169. When CheZΔ138-169 was introduced into various chemotaxis and flagella mutants, it always maintained nearly 100% monopolar localization (Supplementary Figure S2). These results suggest that the role of residues from 138 to 169 on the localization of CheZ_AC_ is not affected by other chemotaxis or flagellar proteins.

**FIGURE 5:**
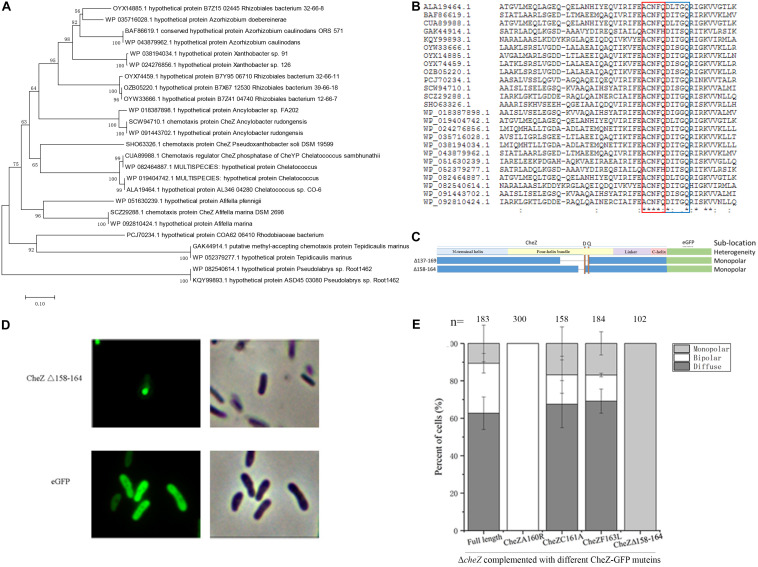
Identification of a novel motif and the effect of some conserved sites of CheZ on polar localization. The phylogenetic tree constructed by twenty-five amino acid sequences of CheZ from species that are closely related to *A. caulinodans* in alpha-*Proteobacteria* **(A)**. Multiple alignment of CheZ protein sequences **(B)**. The position of “ACNFQ” and “DXXXQ” motifs are marked with red and blue boxes, respectively. The construction of a motif (ACNFQ) deleted CheZ mutant **(C)**. The letter “D” and “Q” indicate the position of conserved phosphatase motif DXXXQ. The localization pattern of the novel-motif-deleted mutant **(D)**. The roles of other conserved sites on CheZ sublocalization **(E)**. The “-” means strain containing cognate CheZ derivatives cannot restore chemotaxis.

To find potential conserved sites required for the monopolar localized pattern of CheZ, we analyzed twenty-five amino acid sequences of CheZ proteins from alpha-*Proteobacteria* which are closely related to *A. caulinodans* (Figure 5A). Within residues 138–169, two conserved features were found. One is the conserved phosphatase motif (DXXXQ) (Lertsethtakarn and Ottemann, 2010), and the other is an uncharacterized conserved motif (ACNFQ), which is close to the phosphatase motif (Figures 5B,C). Interestingly, deletion of the ACNFQ motif (CheZΔ158-164) was sufficient to cause the monopolar localized pattern of CheZ_AC_ (Figure 5D). To further investigate whether the monopolar localization resulted from the deletion of ACNFQ, three point-directed mutants, A160R, C161A, and F163L, were constructed successfully. The localization pattern of CheZ_C161A and CheZ_F163L were similar with that of wild-type CheZ_AC_, indicating these residues are not required. CheZ_A160R showed different localization that was nearly 100% bipolar (Figure 5E). These results suggest that the novel motif ACNFQ is involved in the monopolar localization of CheZ_AC_ and conserved site A160 within the motif might contribute more to the function.

### The AXXFQ Motif Is Conserved in *Proteobacteria*

**FIGURE 6:**
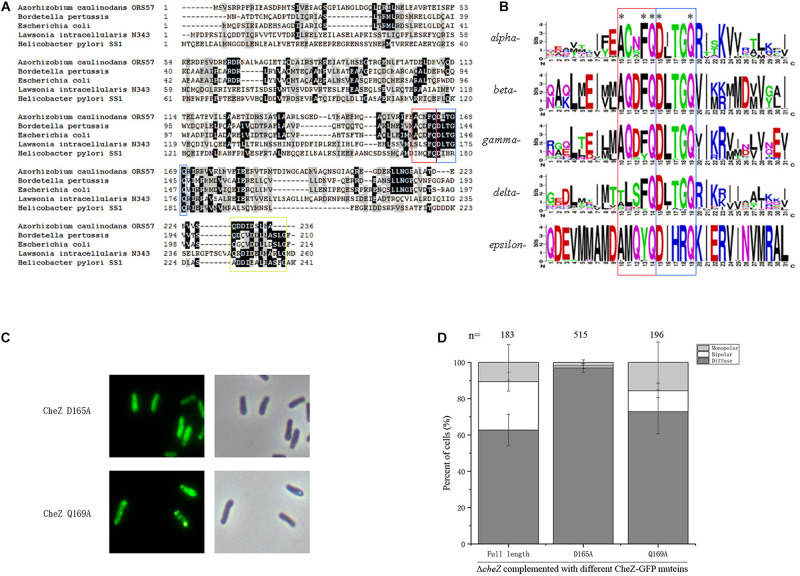
The distribution of AXXFQ motif among *Proteobacteria* and the effect of two key phosphatase active sites, CheZ_D165A and CheZ_Q169A, on CheZ localization. Alignment of five CheZ sequences from representative strains belonging to different *Proteobacteria* **(A)**. Logos of the novel motif built with more than 1800 amino acid sequences among CheZs in *Proteobacteria* **(B)**. The position of “ACNFQ” and “DXXXQ” motifs are marked with red and blue boxes, respectively in **panels A,B**. The yellow box in **panel** **A** means the position of conserved C-terminal region involved in CheY binding. Representative images of localization of CheZ_D165A and CheZ_Q169A **(C)**. Quantification of localization patterns of CheZ_D165A and CheZ_Q169A **(D)**. Data are means ± standard deviation from at least three independent experiments.

CheZ distributes broadly among alpha-, beta-, gamma-, delta-, and epsilon-*Proteobacteria* (Wuichet et al., 2007). Although the degree of identity and similarity between these CheZ proteins are low, the catalytic residues in phosphatase active motif are highly conserved among them (Wuichet et al., 2007). To determine the distribution of the novel motif ACNFQ in *Proteobacteria*, the representative CheZ sequences from each class (alpha-, beta-, gamma-, delta-, and epsilon- *Proteobacteria*) were selected for alignment. All these CheZ proteins have the novel ACNFQ motif close to phosphatase sites (Figure 6A), although the second and third amino acids in the motif are variable among *Proteobacteria*, which was renamed as AXXFQ motif. We then used more than 200 representative sequences from each class to align and build a WebLogo of the conserved region consensus sequences (Crooks et al., 2004). The glutamine residue (Q164 in *A. caulinodans*) near phosphatase sites DXXXQ is the most conserved site among different *Proteobacteria* (Figure 6B). Alanine and phenylalanine residues (A160 and F163 in *A. caulinodans*) are the second conserved sites (Figure 6B). In epsilon-*Proteobacteria*, there is a tyrosine residue instead of phenylalanine (Figure 6B). Considering both tyrosine and phenylalanine have a benzene ring, this might be a conservative substitution. These results showed that the novel motif AXXF(Y)Q is widely distributed and conserved among *Proteobacteria*.

### Phosphatase Active Sites Affect CheZ_AC_ Location

The proximity between these two motifs (AXXFQ and DXXXQ) led us to assess if the localization pattern could be affected by phosphatase active sites. To investigate the role of phosphatase activity on the subcellular localization of CheZ_AC_, site-directed mutants of D165A and Q169A, both critical for the phosphatase activity of CheZ_AC_ (Zhao et al., 2002), were fused to GFP. CheZ_Q169A showed a small increase in diffuse localization, and D165A caused an obvious decrease of polar localization (Figures 6C,D), suggesting the subcellular localization of CheZ_AC_ might be affected by phosphatase active sites. Because the localization of CheZ_AC_ is affected by phosphatase active sites, in turn, the role of different regions affecting localization on chemotactic behavior were assessed. Five truncated mutants, CheZΔ2-31, CheZΔ2-50, CheZΔ2-70, CheZΔ51-70, and CheZΔ71-100, restored the chemotaxis of *cheZ* mutant (Figure 4A and Supplementary Figure S3), suggesting that the N-terminus of CheZ_AC_ is not essential for its phosphatase activity.

## Discussion

The location of CheZ to cell poles can improve the sensitivity to chemotactic stimuli. In *E. coli*, CheZ_F98S, a CheZ_EC_ variant that abolished localization, showed a decreased chemotactic response to external signals (Cantwell et al., 2003; Vaknin and Berg, 2004). Furthermore, the spatial distribution of chemotactic proteins, including CheZ, provides a region for specialized functions which are similar as the membrane-bound organelles in eukaryotic cells (Maddock and Shapiro, 1993). Three localization patterns of CheZ were found in *A. caulinodans*, diffuse, bipolar, and monopolar, indicating the localization pattern of CheZ in *A. caulinodans* is more complex than that in *E. coli* (Sourjik and Berg, 2000; Cantwell et al., 2003). The ratio of cells that demonstrated polar localization of CheZ_AC_ is much lower than that of CheZ_EC_, indicating the role of CheZ_AC_ localization may be different between them. Similar to CheZ in *E. coli*, the localization of CheZ at lateral body of *A. caulinodans* cells showed a typical periodic distribution, and this phenomenon may be interpreted by a “stochastic nucleation model” (Greenfield et al., 2009; Saaki et al., 2018).

For many bacteria, CheZ locates near cell poles where CheY-P is generated, and the phosphatase activity of CheZ is 5- to 10-folds higher at the position (Vaknin and Berg, 2004). The enhanced phosphatase activity of CheZ ensures that peritrichously located flagellar motors experience a uniform concentration of CheY-P, which is critical for the coordinated regulation of flagellar motility (Cluzel et al., 2000; Lipkow et al., 2005; Ringgaard et al., 2011). We determined CheZ_AC_ can still locate to cellular poles despite lacking a CheA binding site (Wuichet et al., 2007) or other chemotaxis or flagellar proteins. The localization of a remote CheZ ortholog in *Helicobacter pylori* has been studied (Lertsethtakarn and Ottemann, 2010; Lertsethtakarn et al., 2015). And, in contrast to *E. coli* CheZ, *H. pylori* CheZ localization is independent of CheA or other typical chemotaxis proteins, but dependent on ChePep, a novel chemotaxis protein distributed among epsilon-*Proteobacteria* (Howitt et al., 2011; Lertsethtakarn et al., 2015). Because the genes encoding homologs of ChePep were not found in *A. caulinodans* genome, we suspect that there might be other partner proteins that contribute CheZ to localization clusters.

The four-helix bundle of CheZ_EC_, especially the tip of the hairpin, is responsible for polar localization in *E. coli*. In this work, we found that the N-terminal helix is sufficient for the polar localization of CheZ_AC_. The sequence conservation of N-terminus of CheZ between *E. coli* and *A. caulinodans* is low, and interestingly, deletion of the N-terminal helix, CheZ_AC_ still remained the polar location, indicating more than one region is sufficient for its polar localization. However, the reason why CheZ_AC_ has two independent regions that are sufficient for polar localization is unknown.

CheZΔ138-169 results in monopolar localization. In this study, a conserved motif AXXF(Y)Q which is close to the phosphatase active motif DXXXQ was found to be responsible for the unique monopolar localized pattern of CheZ_AC_. Although AXXF(Y)Q is conserved among *Proteobacteria*, its role in localization and chemotaxis had not been studied. We speculate the high level of polar localization of CheZ in *E. coli* and *H. pylori* under common conditions may mask the observation of the role of AXXF(Y)Q on localization changes (Sourjik and Berg, 2000; Cantwell et al., 2003; Lertsethtakarn et al., 2015). The biological significance of the polar localization is that each daughter cell can inherit a CheZ after cell division (Jones and Armitage, 2015; Mauriello et al., 2018). For example, the location of chemotactic proteins transfers from monopolar to bipolar clusters in *Vibrio cholerae* before cell division (Ringgaard et al., 2011). The unipolar localization of CheZΔ138-169 or CheZΔ158-164 indicates that one daughter cell cannot inherit CheZ_AC_, and the residues 138–169 might be involved in the dissociation between CheZ_AC_ and its binding partners for localization. In *E. coli*, the polar localization of CheZ_EC_ can be improved by the interaction with CheA at the chemotaxis signaling complex (Wang and Matsumura, 1996; Vaknin and Berg, 2004). These results further suggest that there may be other proteins that recruit CheZ to the clusters and/or affect the catalysis activity of CheZ, as seen in *H. pylori* (Lertsethtakarn and Ottemann, 2010; Howitt et al., 2011; Lertsethtakarn et al., 2015).

In this study, we mapped the critical regions sufficient for CheZ_AC_ localization and assessed the role of regions in the N-terminal helix and four-helix bundle of CheZ_AC_ on both localization changes and chemotaxis. Furthermore, a novel and widespread motif affecting monopolar localization of CheZ_AC_ was identified, which might be also important for the modulation of CheZ polar localization in other *Proterobacteria*.

## Materials and Methods

### Bacterial Strains and Growth Conditions

*Azorhizobium caulinodans* ORS571, its derivatives, and *E. coli* strains are listed in Table 1. *A. caulinodans* strains were grown in TY media at 37°C. *E. coli* strains were cultured in Luria broth (Luria et al., 1960) at 37°C.

**TABLE 1:** Bacteria strains and plasmids used in this study.

**Strain or plasmid**	**Relevant characteristics^a^**	**Source or references**
**Strains**		
*E. coli*		
DH5α	F- *supE44 AlacU169 (ϕ80 lacZ*Δ*M15) hsdR17 recA1 endA1 gyrA96 thi-1 relA1*	Transgen
**Azorhizobium caulinodans**		
*cheZ* mutant	ORS571 derivative; Δ*cheZ*, Amp^R^, Nal^R^, Gm^R^	Liu X. et al., 2018
*cheZ* mutant derivatives	Δ*cheZ* completed with different CheZ variants, Amp^R^, Nal^R^, Gm^R^, Km^R^	This study
**Plasmids**		
pRK2013	Helper plasmid, ColE1 replicon; Tra + Km^R^	Ditta et al., 1980
pBBR1MCS-2	Broad host range plasmid, Km^R^	Kovach et al., 1995
pBBRCheZ	pBBR-1-MCS-2 with *cheZ* open reading frame and 406-bp upstream promoter region; Km^R^	This study
pBBRGFP	pBBR-1-MCS-2 with *egfp* gene; Km^R^	This study
pBBRCheZD165A	pBBR-1-MCS-2 with *cheZ* site substitution mutant and 406-bp upstream promoter region; Km^R^	Liu X. et al., 2018
pBBRCheZQ169A	pBBR-1-MCS-2 with *cheZ* site substitution mutan and 406-bp upstream promoter region; Km^R^	Liu X. et al., 2018
pBBRCheZ-GFP	pBBR-1-MCS-2 with *cheZ* fused with *egfp*; Km^R^	This study
pBBRCheZΔ2-31-GFP	pBBR-1-MCS-2 with *cheZ* lacking bases from 4- 94 bp fused with *egfp*; Km^R^	This study
pBBRCheZΔ2-50-GFP	pBBR-1-MCS-2 with *cheZ* lacking bases from 4- 150 bp fused with *egfp*; Km^R^	This study
pBBRCheZΔ2-70-GFP	pBBR-1-MCS-2 with *cheZ* lacking bases from 4- 210 bp fused with *egfp*; Km^R^	This study
pBBRCheZΔ51-70-GFP	pBBR-1-MCS-2 with *cheZ* lacking bases from 151- 210 bp fused with *egfp*; Km^R^	This study
pBBRCheZΔ71-100-GFP	pBBR-1-MCS-2 with *cheZ* lacking bases from 211- 300 bp fused with *egfp*; Km^R^	This study
pBBRCheZΔ97-137-GFP	pBBR-1-MCS-2 with *cheZ* lacking bases from 292- 411 bp fused with *egfp*; Km^R^	This study
pBBRCheZΔ138-169-GFP	pBBR-1-MCS-2 with *cheZ* lacking bases from 412- 507 bp fused with *egfp*; Km^R^	This study
pBBRCheZΔ71-236-GFP	pBBR-1-MCS-2 with *cheZ* lacking bases from 211- 708 bp fused with *egfp*; Km^R^	This study
pBBRCheZΔ51-236-GFP	pBBR-1-MCS-2 with *cheZ* lacking bases from 151- 708 bp fused with *egfp*; Km^R^	This study
pBBRCheZΔ140-236-GFP	pBBR-1-MCS-2 with *cheZ* lacking bases from 418- 708 bp fused with *egfp*; Km^R^	This study
pBBRCheZΔ170-236-GFP	pBBR-1-MCS-2 with *cheZ* lacking bases from 508- 708 bp fused with *egfp*; Km^R^	This study
pBBRCheZΔ158-164-GFP	pBBR-1-MCS-2 with *cheZ* lacking bases from 472- 492 bp fused with *egfp*; Km^R^	This study
pBBRCheZC161A-GFP	pBBR-1-MCS-2 with *cheZ* with a C161A substitution fused with *egfp*; Km^R^	This study
pBBRCheZF163L-GFP	pBBR-1-MCS-2 with *cheZ* with a F163L substitution fused with *egfp*; Km^R^	This study
pBBRCheZA160R-GFP	pBBR-1-MCS-2 with *cheZ* with a A160R substitution fused with *egfp*; Km^R^	This study
pBBRCheZD165A-GFP	pBBR-1-MCS-2 with *cheZ* with a C161A substitution fused with *egfp*; Km^R^	This study
pBBRCheZQ169A-GFP	pBBR-1-MCS-2 with *cheZ* with a C161A substitution fused with *egfp*; Km^R^	This study

*^*a*^Amp^R^, ampicillin resistance; Gm^R^, gentamicin resistance; Km^R^, kanamycin resistance; Nal^R^, nalidixic acid; Tc^R^, tetracycline.*

### Generation of CheZ Variants

To construct CheZ variants, a fragment including *cheZ* gene and its native promoter was amplified by polymerase chain reaction (PCR). Then an *egfp* gene encoding enhanced GFP was amplified from pEGFP-N1. The two fragments were linked by overlap extension, as previously described (Ho et al., 1989). Next, the resulting construct CheZ-GFP fusion was cloned into a broad-host-range plasmid pBBR1MCS-2 (Kovach et al., 1995), and the pBBR1-CheZ-GFP was used as a temple to construct other CheZ variants. Both the truncated mutants such as CheZΔ2-31-GFP and site-directed mutants such as CheZC161A-GFP were constructed by overlap extension PCR as described by Ho et al. (1989). All the CheZ variants were introduced into the *cheZ* mutant strain by triparental conjugation using a helper plasmid pRK2013 (Ditta et al., 1980). Primer pairs used in the construction are listed in Table 2.

**TABLE 2:** PCR primers used in this study.

**Primers**	**Sequences (5′-3′)^a^**	**Purpose for construction**
cheZ-*Kpn*I-F	GGGGTACC GAAATCACGAGGCCGTAC	pBBRCheZ-GFP, pBBRCheZD165A, and pBBRCheZQ169A construct
gfp-*Xba*I-R	TCTAGATTACTTGTACAGCTCGTC	pBBRCheZ-GFP, pBBRCheZD165A, and pBBRCheZQ169A construct
cheZgfp-R	GCCCTTGCTCACCATGGCGAAGAGGGAGTC	pBBRCheZ-GFP, pBBRCheZD165A, and pBBRCheZQ169A construct
cheZgfp-F	GACTCCCTCTTCGCCATGGTGAGCAAGGGC	pBBRCheZ-GFP, pBBRCheZD165A, and pBBRCheZQ169A construct
CheZΔ2-31-R	ACCGTCGAGCATGCGGTCCGAAGCCT	pBBRCheZΔ2-31 construct
CheZΔ2-31-F	ACCGCATGCTCGACGGTCAACTCGATAGGA	pBBRCheZΔ2-31 construct
CheZΔ2-50-R	TTGAACTTCGACATGCGGTCCGAAGCC	pBBRCheZΔ2-50 construct
CheZΔ2-50-F	CGGACCGCATGTCGAAGTTCAAGGAGCGTGATCCG	pBBRCheZΔ2-50 construct
CheZΔ2-70-R	CGATACCGGCCATGCGGTCCGAAGCCT	pBBRCheZΔ2-70 construct
CheZΔ2-70-F	GACCGCATGGCCGGTATCGAGACGATCC	pBBRCheZΔ2-70 construct
CheZΔ51-70-R	CGATACCGGCGATCTCGGTGCGAACCGC	pBBRCheZΔ51-70 construct
CheZΔ51-70-F	CACCGAGATCGCCGGTATCGAGACGATCCAG	pBBRCheZΔ51-70 construct
CheZΔ71-100-R	GGCGGTGAACCACAAGGCGTTCCGG	pBBRCheZΔ71-100 construct
CheZΔ71-100-F	GCCTTGTGGTTCACCGCCACGGACGAG	pBBRCheZΔ71-100 construct
CheZΔ97-137-R	CCTCGCCGCCGCGGGTGCCC	pBBRCheZΔ97-137 construct
CheZΔ97-137-F	CGCGGCGGCGAGGATCTGACGATGG	pBBRCheZΔ97-137 construct
CheZΔ71-236-R	TGCTCACCATCCACAAGGCGTTCCGGT	pBBRCheZΔ71-236 construct
CheZΔ71-236-F	CGCCTTGTGGATGGTGAGCAAGGGCGAG	pBBRCheZΔ71-236 construct
CheZΔ51-236-R	TTGCTCACCATGATCTCGGTGCGAACCGC	pBBRCheZΔ51-236 construct
CheZΔ51-236-F	GCACCGAGATCATGGTGAGCAAGGGCGAG	pBBRCheZΔ51-236 construct
CheZΔ170-236-R	GCTCACCATCTGGCCGGTGATGTCCT	pBBRCheZΔ170-236 construct
CheZΔ170-236-F	ACCGGCCAGATGGTGAGCAAGGGCGAG	pBBRCheZΔ170-236 construct
CheZΔ158-164-R	CGGTGATGTCAATGCGGACGATCTGCGC	pBBRCheZΔ158-164 construct
CheZΔ158-164-F	CGTCCGCATTGACATCACCGGCCAGCG	pBBRCheZΔ158-164 construct
CheZA160R-R	CCTGGAAGTTGCACCGCTCGAAAATGCGG	pBBRCheZA160R construct
CheZA160R-F	CCGCATTTTCGAGCGGTGCAACTTCCAGG	pBBRCheZA160R construct
CheZC161A-R	GTCCTGGAAGTTGGCCGCCTCGAAAATGC	pBBRCheZC161A construct
CheZC161A-F	GCATTTTCGAGGCGGCCAACTTCCAGGAC	pBBRCheZC161A construct
CheZF163L-R	CGGTGATGTCCTGTAAGTTGCACGCC	pBBRCheZF163L construct
CheZF163L-F	GGCGTGCAACTTACAGGACATCACCG	pBBRCheZF163L construct

*^*a*^Engineered restriction sites are underlined.*

### Microscopy and Data Analysis

After growing in TY solid medium for overnight with shaking, cells with GFP fusion were used for observation. Agarose pads were used to immobile bacteria as described by Meier and Scharf (2009). Images were taken by an Olympus DP73 camera on an Olympus BX53 system fluorescence microscope with a 100 × objective and controlled by a cellSens Dimension 1.7 imaging software (Olympus Inc.,). A space between 505 to 550 nm filter was used to detect fluorescence signals. The images analyzing spatial distribution of CheZ were processed by ImageJ^1^ as described by Thiem et al. (2007). Distribution of CheZ_AC_ was manually enumerated and classified into three types (diffuse, bipolar, and monopolar localization). CheZ_AC_ cells with monopolar or bipolar localization showed obvious bright spots at one end or both ends of cell. When the brightness in the whole cell distribute evenly, the localization of CheZ_AC_ in these cells is counted as diffuse. ImageJ was used to quantify the brightness at different regions of cells. Experiments were repeated at least three times, and for each sample at least 100 cells were counted.

### Soft Agar Plate Assay

The chemotactic behavior of *cheZ* mutant derivative strains was assessed using soft agar plate assay, as previously described (Miller et al., 2009). Overnight bacterial cultures were washed with chemotaxis buffer at least two times and then adjusted to OD_600_ of 0.6. Five microliter of cells was dropped in the center of 0.3% soft agar plate. After culturing for 3 days at 37°C, the chemotactic rings on soft agar plate were counted. Ten mM sodium lactate was used as sole carbon source. Experiments were repeated at least three times.

### Sequence Alignment and Analysis

CheZ sequences from different *Proteobacteria* classes were selected from Mist 2.2^2^ (Ulrich and Zhulin, 2010) and NCBI database^3^. The amino acid sequences alignment was performed by an EMBOSS Needle program^4^. Secondary and tertiary structure of CheZ proteins were predicted using online server SWISS-MODEL^5^ and Jpred4^6^. The phylogenetic tree was established using MEGA7 software (Kumar et al., 2016). The multiple sequences of CheZ were aligned by T-coffee^7^ (Notredame et al., 2000) and BioEdit (Alzohairy, 2011). Hundreds of CheZ sequences selected from NCBI database from each class of *Proteobacteria* were aligned by MEGA7, then the constructing file was put into Jalview (Waterhouse et al., 2009) to produce a graphical representation, and the region including phosphatase active sties was put into WebLogo (Crooks et al., 2004).

## Data Availability Statement

The raw data supporting the conclusions of this article will be made available by the authors, without undue reservation.

## Author Contributions

XL and ZX conceived and designed the experiments, analyzed the data, prepared the figures and tables, and wrote the manuscript. XL, YL, and XD carried out the experiments. KJ helped with the improvement and revision of the manuscript. All authors approved the submission for publication.

## Conflict of Interest

The authors declare that the research was conducted in the absence of any commercial or financial relationships that could be construed as a potential conflict of interest.

## Footnotes

1 https://imagej.nih.gov/ij/

2 https://mistdb.com/

3 https://www.ncbi.nlm.nih.gov/

4 https://www.ebi.ac.uk/Tools/psa/emboss_needle/

5 https://swissmodel.expasy.org/

6 http://www.compbio.dundee.ac.uk/jpred/

7 https://www.ebi.ac.uk/Tools/msa/tcoffee/

## Supplementary Material

Supplementary Figure 1 The alignment of CheA_EC_ and CheA_AC_ was generated using the default settings of EMBOSS Needle software from EMBL. The beginning Met of the short form of CheA_EC_ was marked with black frame.

Supplementary Figure 2 The localization pattern of CheZΔ138-169 in *cheA*, *cheA-R*, *cheY1*, *cheY2*, *fliM*, and *fliN* mutant. The mutein CheZΔ138-169 was fused to GFP, and CheZΔ138-169GFP including its own promoter were inserted into pBBR2 and then were transformed into each mutant strain.

Supplementary Figure 3 The representative images of chemotactic rings formed by *cheZ* mutant containing different CheZ derivatives fused to GFP. Ten mM sodium lactate was used as sole carbon source. The cognate results of each strain also shown in Figures 4A and 5A.
