# Supplementary figures and images for: Protein Residues and a Novel Motif Involved in the Cellular Localization of CheZ in Azorhizobium caulinodans ORS571
Source: Front Microbiol. 2020 Dec 7;11:585140. doi: 10.3389/fmicb.2020.585140 (PMC7750401; doi:10.3389/fmicb.2020.585140)

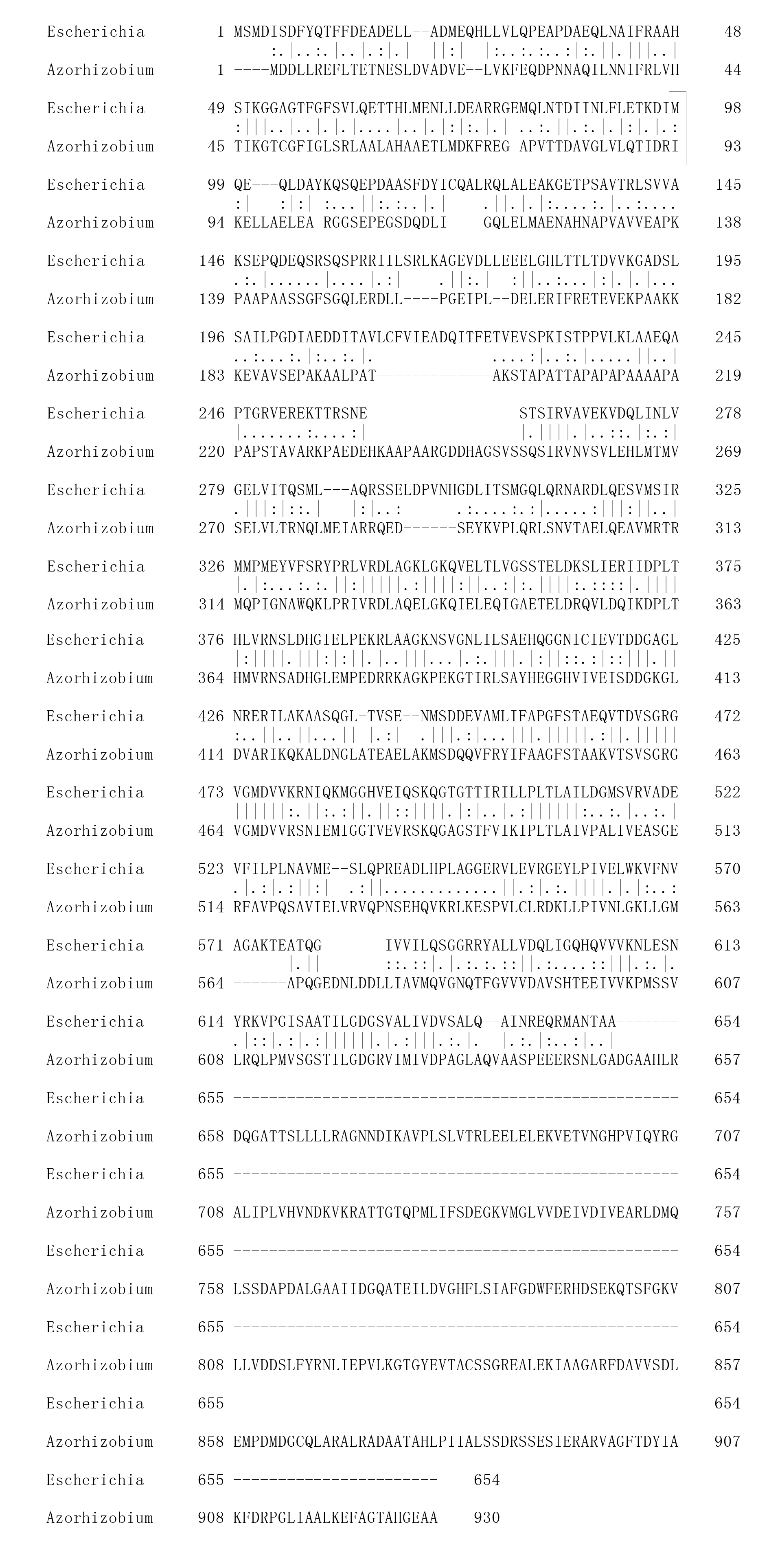

Supplement: Supplementary Figure 1 — The alignment of CheAEC and CheAAC was generated using the default settings of EMBOSS Needle software from EMBL. The beginning Met of the short form of CheAEC was marked with black frame. [file Image_1.TIF]

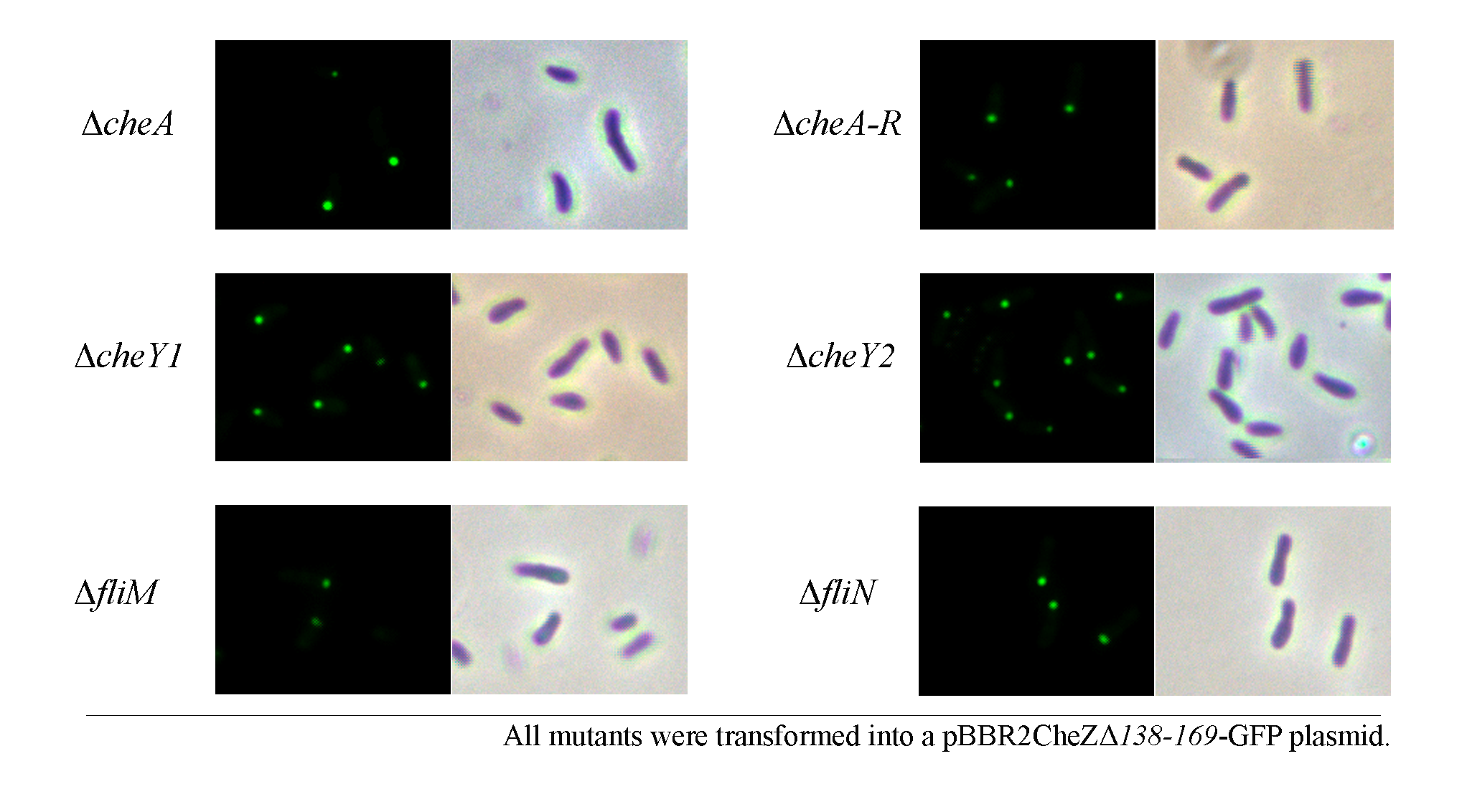

Supplement: Supplementary Figure 2 — The localization pattern of CheZΔ138-169 in cheA, cheA-R, cheY1, cheY2, fliM, and fliN mutant. The mutein CheZΔ138-169 was fused to GFP, and CheZΔ138-169GFP including its own promoter were inserted into pBBR2 and then were transformed into each mutant strain. [file Image_2.TIF]

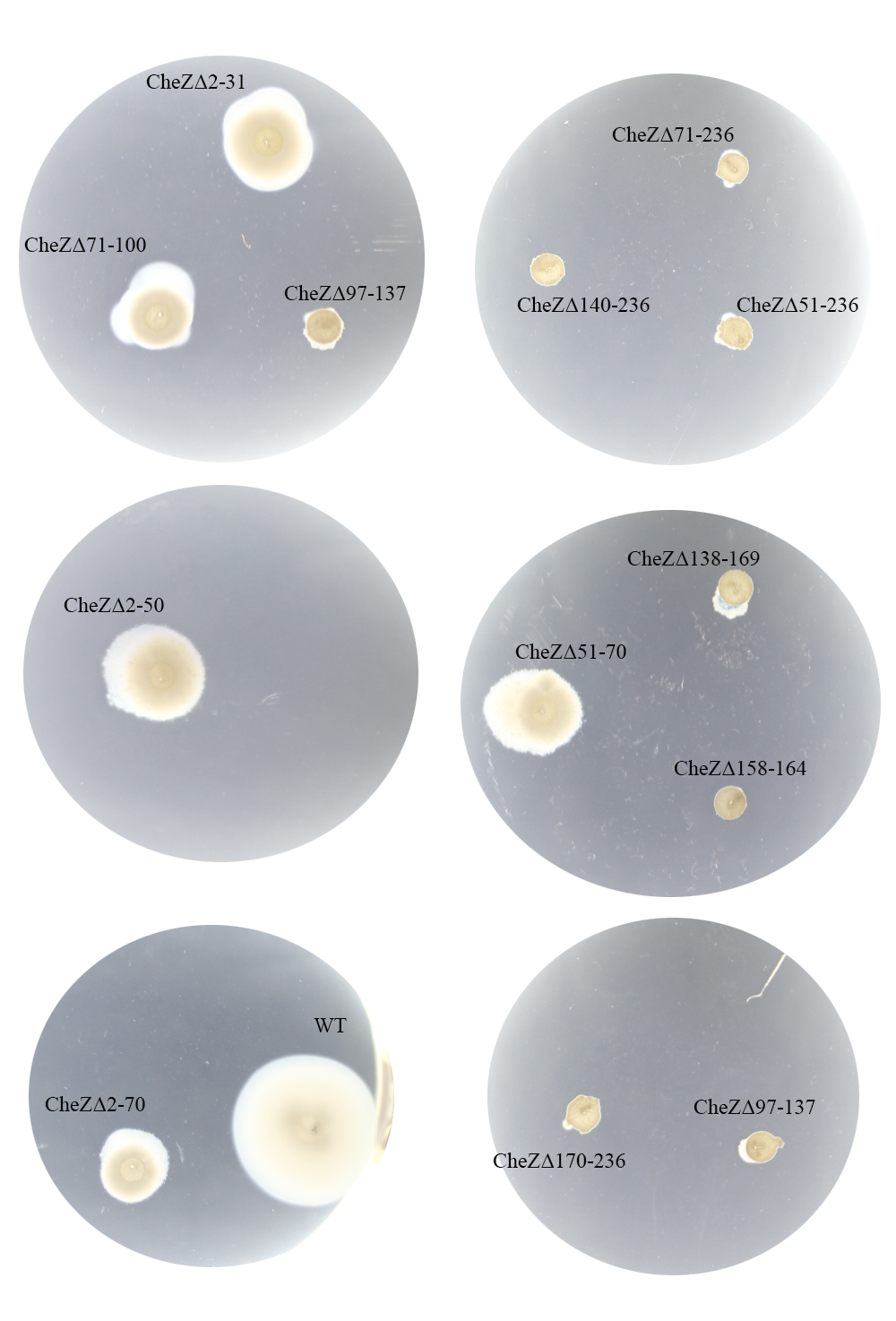

Supplement: Supplementary Figure 3 — The representative images of chemotactic rings formed by cheZ mutant containing different CheZ derivatives fused to GFP. Ten mM sodium lactate was used as sole carbon source. The cognate results of each strain also shown in Figures 4A and 5A. [file Image_3.TIF]
